# Novel Micro- and Nanocellulose-Based Delivery Systems for Liposoluble Compounds

**DOI:** 10.3390/nano11102593

**Published:** 2021-10-01

**Authors:** Francisca Casanova, Carla F. Pereira, Alessandra B. Ribeiro, Ricardo Freixo, Eduardo Costa, Manuela E. Pintado, João C. Fernandes, Óscar L. Ramos

**Affiliations:** CBQF—Centro de Biotecnologia e Química Fina—Laboratório Associado, Escola Superior de Biotecnologia, Universidade Católica Portuguesa, Rua Diogo Botelho 1327, 4169-005 Porto, Portugal; fcbastos@ucp.pt (F.C.); abribeiro@porto.ucp.pt (A.B.R.); rfreixo@ucp.pt (R.F.); emcosta@ucp.pt (E.C.); mpintado@ucp.pt (M.E.P.); jcfernandes@porto.ucp.pt (J.C.F.)

**Keywords:** cellulose, microcrystalline cellulose, nanocellulose, delivery systems, liposoluble compounds

## Abstract

Poor aqueous solubility of bioactive compounds is becoming a pronounced challenge in the development of bioactive formulations. Numerous liposoluble compounds have very interesting biological activities, but their low water solubility, stability, and bioavailability restrict their applications. To overcome these limitations there is a need to use enabling delivering strategies, which often demand new carrier materials. Cellulose and its micro- and nanostructures are promising carriers with unique features. In this context, this review describes the fast-growing field of micro- and nanocellulose based delivery systems with a focus on the release of liposoluble bioactive compounds. The state of research on this field is reviewed in this article, which also covers the chemistry, preparation, properties, and applications of micro- and nanocellulose based delivery systems. Although there are promising perspectives for introducing these materials into various fields, aspects of safety and toxicity must be revealed and are discussed in this review. The impact of gastrointestinal conditions on the systems and on the bioavailability of the bioactive compounds are also addressed in this review. This article helps to unveil the whole panorama of micro- and nanocellulose as delivery systems for liposoluble compounds, showing that these represent a great promise in a wide range of applications.

## 1. Introduction

**Figure 1:**
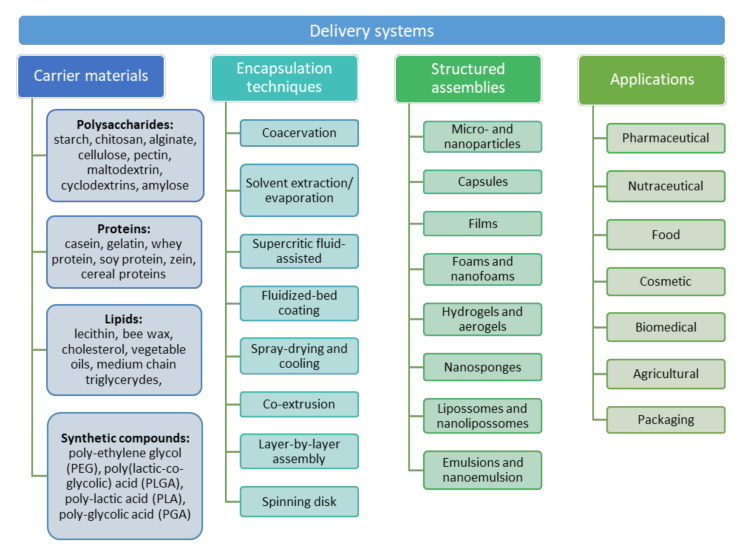
Systematic overview of encapsulating materials, techniques, structured assemblies, and applications of delivery systems.

Controlled delivery technology represents a widely studied area in the field of pharmaceutical [1], nutraceutical [2], food [3], and cosmetic sciences [4]. The main aim when formulating delivery systems is to preserve and enhance the bioavailability of bioactive compounds. Delivery systems allow the improvement of the stability, biological activity, and bioavailability of bioactive compounds, as well as their controlled and targeted release. Moreover, they permit the reduction of toxicity and side effects, elimination of a specific administration route, reduction in dosing frequency, and improvement in consumer convenience [5,6,7]. Different systems with a variety of carrier materials and produced by several techniques have been developed to control the temporal and spatial release profile of bioactive compounds (Figure 1). The design of the system and the choice of materials and techniques depend on the particular application, the desired releasing profile and the properties of the bioactive compound to be delivered [7,8].

Delivery systems at micro- and nanoscale have attracted considerable interest worldwide over the past years. The European Food Safety Authority (EFSA) refers to nanomaterials as engineered materials that have at least one dimension in the range of 1–100 nm [9], while the International Union of Pure and Applied Chemistry (IUPAC) defines micromaterials as materials with sizes between 1 and 1000 μm [10]. The main benefit of using micro- and nanomaterials as delivery systems is due to the fact that the properties of materials change when their scale is reduced, resulting in distinctive physicochemical and biological characteristics. The bioavailability of loaded bioactive compounds often increases when the size of the particles decreases, namely due to faster digestion, ability to penetrate the mucus layer, or even by direct uptake by cells. These systems can also often release their payload at a specified point, thus maximizing their potential health benefits [11].

A high number of interesting bioactive compounds which have promising biological functions and health-promotional effects are liposoluble, meaning that they exhibit low water solubility, stability, and bioavailability. This results in unsatisfactory efficacy when administrated to the body and restricts their application in the pharmaceutical, nutraceutical, food, and cosmetic fields [12,13,14,15]. A number of advancements have been made in the past 30 years in the development of new techniques and materials for controlled delivery [6,16,17]. To formulate delivery systems with desirable properties and releasing profiles, the constant improvement of existing materials and the creation of new ones with a broad variety of physicochemical properties constitutes an imperative demand [8].

Considerable attention has been drawn to natural polysaccharides as carrier materials, due to their abundance, low cost, low toxicity, biocompatibility, and biodegradability. Cellulose, the most abundant natural polymer, and its micro- and nanostructures, namely microcrystalline cellulose (MCC), cellulose nanocrystals (CNC), and cellulose nanofibers (CNF), are promising carriers due to their unique features, as further discussed in this review. The successful extraction of cellulose and its derivatives from a broad range of lignocellulosic biomass has greatly demonstrated their promising renewability and sustainability, providing a substantial environmental advantage compared with other materials [5,18]. Industrial production of micro- and nanocelluloses is increasing rapidly with several companies, e.g., DuPont (United States), Celluforce (Canada), Innventia (Sweden), and Nippon Paper Group (Japan), already producing on the tons-per-day scale, intensifying the quest for viable products across many sectors.

**Figure 2:**
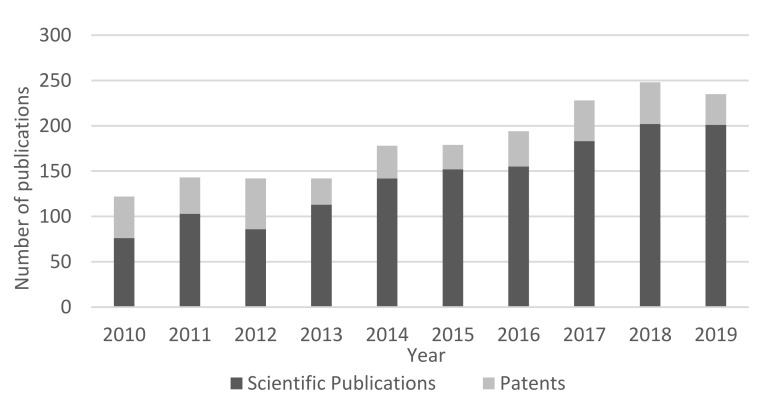
Literature survey of published research articles and patents in cellulose for controlled delivery, using a search query with keywords “cellulose” and “controlled delivery” or “cellulose” and “controlled release”, from 2010 to 2019 via Web of Science™ and WIPO (World Intellectual Property Organization).

In this line, the number of publications referring to the use of cellulosic materials as controlled delivery systems has known an interesting growth over the last decade (Figure 2). To date, a total of ca. 2000 scientific publications and 700 patents related to this topic are available on international databases, of which ca. 1500 and 400, respectively, were published in the last decade. The interest in this topic has known a significant increase and about 40% of this decade’s scientific papers were published in the last 3 years. These studies have been conducted for a variety of applications, including cancer therapy [19], antibacterial effect [20,21], packaging [22], wound healing [20,23], transdermal delivery [24,25,26], agro-chemistry [27], and delivery of a variety of drugs [28,29].

Although there are promising perspectives for introducing these materials into delivery systems for various applications, prior to achieving commercial products there are still some questions that need to be addressed. Challenges encompass understanding the behavior of these delivery systems inside the body in terms of safety and toxicity, but also issues regarding the digestion, absorption, and biodegradation of such systems, as well as the influence on bioaccessibility and bioavailability of the bioactive compounds to be delivered.

This review gives a broad overview of cellulose micro and nanostructures capabilities and applications for the delivery of liposoluble compounds. The unique properties of these structures, as well as their sources and isolation methods from lignocellulosic biomass, are described. Studies on the development and application of innovative cellulose-based materials for the delivery of liposoluble compounds were collected and are herein discussed. There are already some reviews on the applications of cellulose materials for controlled delivery. For instance, Sun et al. [30] reviewed the applications of cellulose-based materials in sustained drug delivery systems, Plackett et al. and Xie & Li [14,31] compiled reviews on nanocellulose as a novel carrier for drug delivery, and Seabra et al. [32] focused on cellulose nanocrystals as carriers in medicine. However, there is a lack of compiled and integrated information on the use of cellulose structures (at the micro and nanoscale) targeted to the delivery of liposoluble compounds, for various applications. This review discusses and highlights the most promising of these structures, and it further goes beyond the existing ones by dealing with hot topics in the field, such as potential toxicity and how such systems behave under GI conditions, addressing bioaccessibility, bioavailability, and digestibility.

## 2. Cellulose-Based Materials

### 2.1. Cellulose Basics: Sources, Isolation Methods, and Structural Features

Cellulose is the world’s most abundant natural polymer, an almost inexhaustible source of raw material, representing 1.5 × 10^12^ tons of the planet’s total annual biomass production [33]. It is biodegradable, biocompatible, and renewable, being an alternative to non-degradable fossil-fuel-based polymers [34,35]. The most commercially exploited natural resource containing cellulose is wood (hardwood pulp and softwood pulp) [36,37,38], but non-wood plant fibers, such as kenaf [39], sisal [40], jute [41], sugarcane [42,43,44] and cotton [38,45,46], represent cellulose sources with great potential. Furthermore, there are also non-plant sources of cellulose, namely cellulose produced by bacteria [47], algae [48], and tunicates [49]. Cotton has the highest cellulose content of plants, with about 90% cellulose, compared to wood (40–50% cellulose content) or bast fibers, such as kenaf, sisal, and jute (70–80% cellulose content) [50,51,52]. In the context of sustainable development, lignocellulosic biomass from industrial and agricultural wastes has attracted much attention as cellulose sources. These bio-residues have an advantage compared to other cellulosic feedstocks by having high availability, low or even no costs, and possibly contributing to solving disposal problems for industries. Their use is desirable from an environmental point of view and permits the increase of the value of underutilized renewable materials [43,50].

Different methods have been described for the isolation of cellulose from these sources, having the purpose to remove the accompanying materials, e.g., lignin and hemicellulose. The choice of cellulose extraction method depends on the cellulose plant source, desired fiber dimensions, required purity and yield, both of which depend on the further application of the obtained cellulose [53]. Methods such as: (i) alkaline treatment [54,55]; (ii) acid treatment [56]; (iii) bleaching [57]; (iv) ionic liquid extraction [58,59]; (v) microwave extraction [60]; (vi) ultrasonic extraction [61]; (vii) enzymatic treatment [62]; and (viii) combinations thereof [63,64], have been described for cellulose extraction and were comprehensively reviewed by Radotić & Mićić [53]. Although these methods have been extensively reported in the literature, they possess many limitations that restrict their development and application in the industry. Acid and alkaline treatments pose problems in environmental pollution (harsh chemicals), safety, and fiber damage; physical methods (microwave, ultrasonic, steam explosion) have a high-energy consumption (environmental impact) and use expensive devices; and enzymatic processes are high-cost, time-consuming and only moderately efficient [65]. With sustainable development becoming top of the agenda, researchers are continuously looking for greener, more efficient, and safer methods for cellulose extraction. Methods that have recently attracted much attention include: (i) extraction with deep eutectic solvents [66], (ii) organosolv [67], (iii) autohydrolysis [68,69], and (iv) supercritical extraction [70].

**Figure 3:**
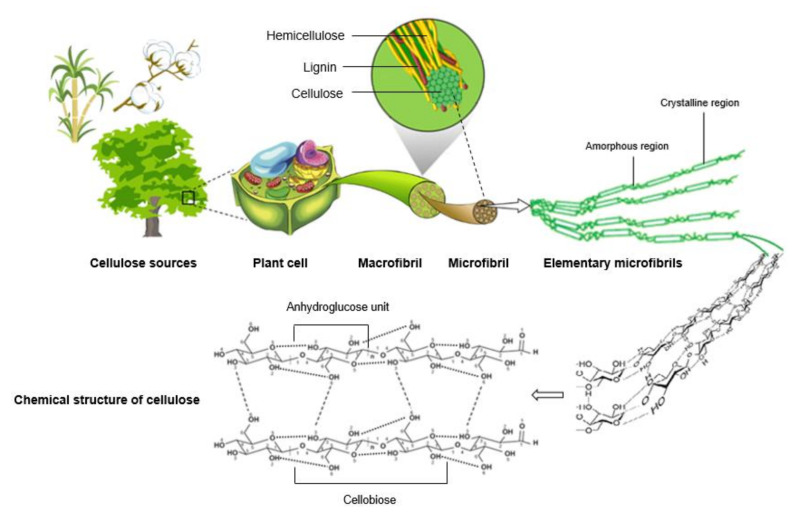
Schematic representation of the hierarchical structure of cellulose fibers and the chemical structure of cellulose.

The chemical structure of cellulose (Figure 3) shows that the polymer is composed of anhydroglucose units (AGU) linked together by β-1,4-glycosidic bonds, forming a high–molecular-weight linear homopolymer, of which cellobiose is the smallest repeating unit in the polymer. The degree of polymerization (DP)—a measure of how many AGUs there are in the polymer, has an important influence on the fiber properties. One of the most specific characteristics of cellulose is that each of its internal AGU bears three hydroxyl groups, which provide cellulose structures with a reactive surface covered with numerous active groups. The ability of these hydroxyl groups to establish hydrogen bonds plays an important role in the formation of fibrillar and semi-crystalline structures, governing the characteristic physical properties of these highly cohesive materials [71,72,73]. Comprehension of cellulose association inside plants is vital for effective understanding of the properties of micro and nanocellulose structures, as well as for the development of their production procedures [74]. The hierarchical structure of cellulose fibers is also shown in Figure 3. Cellulose molecules are packed together in parallel into semi-crystalline microfibrils that are held together via inter- and intramolecular hydrogen bonds and Van der Waals forces. Cellulose microfibrils are characterized by a diameter range from 2 to 30 nm, depending on cellulose source, and a length that can be of several micrometers. These microfibrils are composed of both crystalline and amorphous regions and assemble into larger units called macrofibrils (Figure 3), which in turn are further arranged into macroscopic plant cellulosic fibers, where the cellulose fibrils are embedded in a soft matrix mainly composed of lignin and hemicellulose [7,12,50].

Cellulose materials, being highly ordered bundles of cellulose chains aligned along the bundle axis, exhibit a unique combination of physical properties. These include superior mechanical properties, flexibility, elasticity, low thermal expansion, high thermal stability, good rheological properties, high optical transparency, and relatively low density (1.6 g/cm^3^) [52,73]. The mechanical properties of cellulose materials are determined by their properties in both the crystalline (ordered) and amorphous (disordered) regions [73]. Cellulosic chains in amorphous regions provide flexibility and plasticity to the material, while those in crystalline regions provide stiffness and elasticity [75]. The organization of cellulose molecules results in high anisotropy; i.e., the properties transverse to the cellulose chains are different (usually lower) than the properties in the direction of the chains [52].

As stated, the hydroxyl groups impart cellulose some of its characteristic properties, such as hydrophilicity, chirality, hierarchical organization, and high cohesion [34]. These are due to their high reactivity and ability to form strong hydrogen bonds, which also makes them possible sites for further chemical modification in order to tune cellulose properties, namely solubility, as cellulose is mainly soluble in harmful solvents that are difficult to remove [7,34,51]. Typical modifications of cellulose include etherification, esterification, sulfonation, silylation, amidation, depolymerization (oxidative or hydrolytic), and radical grafting (cationic/anionic modification). Comprehensive reviews on this topic were recently done by Sun et al. and Rol et al. [30,76]. The surface chemistry of cellulose structures is critically important in determining the interaction between the materials and their environment, their dispersion in solvents or polymers, rheological properties, self-assembly, agglomeration, interfacial interactions and, in the specific case of delivery systems, the duration and destination of these materials within the body [52].

### 2.2. Cellulose Supramolecular Structures

**Figure 4:**
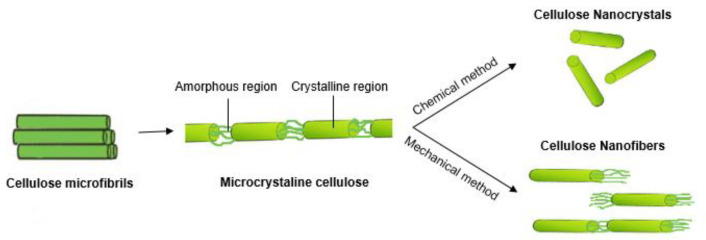
Microcrystalline cellulose (MCC) and nanocellulose (CNC and CNF) production from cellulose microfibrils.

The structure of native cellulosic fibers results in two main families of materials: microcrystalline cellulose (MCC) and nanocellulose (NC), which can be further divided into cellulose nanocrystals (CNC) and cellulose nanofibers (CNF) (Figure 4). These have essentially different extraction procedures as well as different dimensions, morphologies, and crystalline structures, which will be further detailed in the next subsections. According to ISO/TS 20477:2017, nanocellulose is a material composed predominantly of cellulose with any external dimension in the nanoscale: from 1 nm to 100 nm, while microcellulose is a material composed predominantly of cellulose with any external dimension in the microscale. Nonetheless, there are several inconsistencies in the description of the different sets of cellulose materials and several terms coexist in the literature [77]. This review will use as definition for CNC as crystals with a diameter of 3–10 nm and length between 15 and 500 nm; CNF as fibers with a diameter of 5–30 nm and length up to several micrometers; and MCC as particles and/or fibers of 10 to 50 μm, according to the WI 3021 standard proposed by The Technical Association of the Pulp and Paper Industry [78].

The morphology and dimension of cellulose micro- and nanostructures can be examined using advanced light-scattering systems and microscopy techniques, such as Transmission Electron Microscopy (TEM), Scanning Electron Microscopy (SEM), and Atomic Force Microscopy (AFM). These structures are usually further characterized by structural, elemental, and thermal analysis, using Fourier Transform Infrared Spectroscopy (FT-IR), X-ray diffraction analysis (XRD), Nuclear Magnetic Resonance Spectroscopy (NMR), Thermogravimetric Analysis (TGA) and Differential Scanning Calorimetry (DSC) [43,79].

Micro and nanocellulose, being natural micro and nano-sized materials, contain various beneficial features. They have unique morphologies and geometrical dimensions, high crystallinity, high specific surface area, high aspect ratios (length/width), barrier properties, mechanical reinforcement properties, good rheological properties, surface chemical reactivity, and accessible hydroxyl groups that can be chemically modified to give additional functionalities [52]. Furthermore, these have the ability to bind both polar and non-polar bioactive compounds due to their hydrophilic and hydrophobic character [40,41]. Nevertheless, MCC, CNC, and CNF differ in their properties due to differences in morphology, dimensions, and structure [12]. Furthermore, physicochemical properties and yield of cellulose micro and nanostructures are strongly associated with the chemical composition of their plant sources and the processing methods adopted for pre-treatment and extraction [50,52].

Cellulose micro and nanostructures represent inspiring advances in cellulose science, technology, and product development for the next generation of renewable and sustainable products. Whereas the bottleneck for technology deployment of many micro and nanoscale materials is scalable manufacturing, cellulose is produced daily by approximately 3,000,000,000,000 trees and other plants, such as fast-growing bamboo and sugarcane. Therefore, cellulose based-materials provide a nearly unlimited resource for functional sustainable materials in a wide range of applications [73].

#### 2.2.1. Microcrystalline Cellulose—MCC

Microcrystalline cellulose (MCC) is a purified, partially depolymerized cellulose, usually obtained via hydrolysis of cellulose fibers. The obtained MCC consists of agglomerates of pure α-cellulose isolated as a white, odor, and tasteless powder. The degree of polymerization (DP) is used as an identity test, as pharmacopoeial MCC is defined by a DP below 350 glucose units, which contrasts to DPs in the order of 10,000 units for the native cellulose. The size of MCC particles depends on the source and processing conditions but is usually between 5 and 50 μm [80,81].

MCC can be synthesized by different processes, including chemical (e.g., acid and alkaline hydrolysis, organosolv), mechanical (e.g., steam explosion, extrusion), and biological (e.g., enzymatic hydrolysis) methods as reviewed by Trache et al. [82]. From these, the acid hydrolysis process constitutes the most preferable pathway due to its lower cost, shorter duration and possibility to be applied as a continuous process rather than a batch-type process. Furthermore, this process usually obtains smaller particles of MCC as the final product. In this method, fibrous plant pulp is hydrolyzed by a mineral acid, traditionally H₂SO₄ or HCl, under heating at 45–120 °C. The amorphous phase is readily hydrolyzed when subjected to acid hydrolysis, which results in shorter and more crystalline fragments [82]. Effective parameters in the acid hydrolysis process to produce MCC include: acid type and concentration, acid/fiber ratio, hydrolysis time, and hydrolysis temperature. The most well-established method, and the one commonly used to obtain MCC as a carrier for delivery systems, involves acid hydrolysis with HCl 2.5 N at reflux temperature (105 °C) for 15 min [83,84,85]. Further filtration and mechanical treatments, such as homogenization and sonication, have been employed for size modulation [84,85,86].

MCC is a traditional excipient in pharmaceutical, food, and cosmetic formulations, being approved by the European Food Safety Authority (E-number: E460(i)) and the U.S. Food and Drug Administration (FDA) for use as an additive in food products [51]. It has been widely used as filler for tablet production [80], and is an option for delivery purposes [7,30]. Several commercial forms of MCC, such as Avicel^®^, Pharmacel^®^, Ceolus^®^, Celphere™, and Ethispheres^®^ in a variety of grades are now available [87,88,89,90]. Avicel^®^ and MCC spheres, such as Celphere™ and Ethispheres^®^, have been the most used in liposoluble compound delivery studies [91,92,93,94].

#### 2.2.2. Cellulose Nanocrystals—CNC

Since the natural cellulose microfibrils consist of both amorphous and crystalline regions, treatment of them in acidic conditions leads to extensive hydrolysis of the amorphous fractions and formation of short rod-shaped cellulose nanoparticles with high crystallinity degree and low aspect ratio (length/width). CNCs may have sizes ranging from 3–10 nm width by 15–500 nm length and 5–50 aspect ratio. The crystallinity indices typically of 70–90% are dependent on the source material and process conditions. Several terms are used in the literature to denote these crystals, such as cellulose nanocrystals, nanowhiskers, nanorods, and nanocrystalline cellulose [7,15,95,96], being cellulose nanocrystals (CNC) the most frequently used and therefore the terminology adopted in this review. Since the discovery of CNC in 1949 by Bengt G. Rånby [97], innumerous potential applications have been found, as reviewed by Grishkewich et al. [98]. Recent interest in the application of CNC as a biomaterial, namely in delivery systems, as carrier [39], cross-linking agent [99], and filler [100] has grown as a result of its crystalline proprieties, high specific surface area, biocompatibility, and biodegradability [15,95,99].

CNC can be obtained by extraction of crystalline cellulosic regions through a variety of processes [96]. Chemical methods such as: (i) acid hydrolysis [42,43,44,79,101,102,103,104]; (ii) TEMPO mediated oxidation [105]; (iii) ammonium persulfate oxidation [106]; (iv) ionic liquids and eutectic solvents extraction [37,107,108,109]; as well as biological methods: (i) enzymatic [110] and (ii) microbial [111] hydrolyses have been reported. Combinations of methods, such as acid hydrolysis in the presence of oxidizers (e.g., H_2_O_2_), have also been described [65]. However, a well-known process based on acid hydrolysis is generally utilized, namely to produce CNC applied to the delivery of liposoluble compounds, which conditions and resulting CNC properties are shown in Table 1. The most common reaction conditions involve the utilization H_2_SO_4_ (64% *w/w*) at 45 °C for 40–45 min [28,39,95]. For the same acid hydrolysis conditions, studies that use sonication as mechanical after-treatment are able to achieve CNC with smaller dimensions (e.g., 10 nm width vs. 50 nm) and higher degrees of crystallinity (e.g., 90% vs. 70%) [44,54]. However, the possible influence of different cellulose sources in this comparison should not be overlooked. Acid hydrolysis by H_2_SO_4_ results in the functionalization of the surface of CNCs with negative sulfate groups (–OSO_3_-), which leads to a well-dispersed stable colloidal suspension in water, making CNCs useful for biological applications [15]. CNCs prepared with other acids, such as HCl [112] or HBr [113], will not have any surface charges, and a stable dispersion is, therefore, harder to form. Furthermore, from an industrial level, H_2_SO_4_ is the most suitable choice, since it is one of the most abundant and economically produced chemicals in the world [51].

Nanocellulose is nowadays an available product from various companies and research institutes around the world. The first and most significant commercial development of CNC is CelluForce, which, based upon research at FP Innovations (Canada), started manufacturing CNC in bulk (CelluForce NCC™) in 2012. Nowadays, several CNC products from different companies are available, e.g., from Rettenmeier & Söhne (Germany) and Cellulose Lab (Canada), and the Global Market Insights Inc. has claimed it will be a USD 40 billion industry by 2024 [7,51].

#### 2.2.3. Cellulose Nanofibers—CNF

When the macroscopic cellulose fibers are mechanically disintegrated, avoiding the strongly acidic conditions, long nanoscale fibrils that contain both crystalline and amorphous regions are produced. These fibers have typically high aspect ratios (length/width), usually greater than 50. In the literature terms such as cellulose nanofibers (CNF), cellulose nanofibrils, nanocellulose fibers/fibrils or nanofibrillated cellulose have been used to describe these materials [7,12,118], where cellulose nanofibers are the most frequently employed and therefore the terminology adopted in this review. Owing to its excellent mechanical properties, CNF has been extensively explored in various fields, such as material sciences [73], composites [119], packaging [120], paper [121], and catalysis [122]. CNF has also emerged as candidates for biomedical/pharmaceutical [12,123] and cosmeceutical/nutraceutical [124] applications, namely as delivery systems [8,36]. Its distinctive physicochemical properties at different interfaces and large surface-area-to-volume offer possibilities for positive molecular interactions with active molecules, stabilization of particles and suspensions, modification of rheological properties, improvement of the mechanical stability of dosage forms, and formation of nanoparticles embedded aerogels [7,8,12,125].

CNF can be produced through different methods, but all are based on the separation of the fibers while keeping their amorphous parts intact. In these processes, microfibril strands from cellulose fibers are peeled off by high shearing forces that cleave the macroscopic cellulose structures along the longitudinal axis of the cellulose microfibrillar structure, resulting in a long and soft nanosized (in diameter) chain. The length of the nanofiber will be highly dependent on the exposition degree of the material to mechanical processing and the final product is a suspension with the appearance of a highly viscous gel [7,12,74]. Most of the methods are therefore mechanical processes, such as: (i) high pressure homogenization [126,127,128]; (ii) microfluidization [37,129,130]; (iii) grinding [128,131]; (iv) cryocrushing [132,133] and (v) ultrasonication [134]. These are often coupled with chemical pre-treatments, such as: carboxymethylation [135,136], TEMPO-mediated oxidation [38,137,138], acetylation [139] and alkali pretreatment [132,133], or even with biological (enzymatic) pretreatments [140,141,142], which facilitate the release of more individualized CNF and decrease the energy demanded for the procedure [7,50,143]. The most common method for the production of CNF to be used as a carrier for liposoluble compounds (Table 1) utilizes high-pressure homogenization at 1650 bar [116,117], probably because these devices are easily scalable for use at an industrial level. CNF commercial products are currently widely available, namely from companies such as UPM–Kymmene Corporation (Finland), Stora Enso (Finland), Daicel (Japan), Innventia (Sweden), and Nippon Paper Group (Japan). Mostly, commercial grades of CNF from UPM-Kymmene have been used for the controlled release of liposoluble compounds [118,144,145].

## 3. Challenges of Liposoluble Compounds Delivery

Poor aqueous solubility of bioactive compounds in pharmaceutical, nutraceutical, and cosmeceutical industries is becoming an increasingly pronounced challenge in the development of bioactive formulations. Around 40% of marketed active compounds and up to 70% of candidates showing high potential in the pipeline of these industries show hydrophobicity, liposolubility, or poor aqueous solubility, consequently resulting in unsatisfactory biological efficacy when administrated, due to inconsistent GI absorption [12,13,14,15]. Bioactive compounds such as lipophilic phenols, carotenoids, phytocannabinoids, essential fatty acids, lipophilic vitamins (A, D, E, and K) or phytosterols have very interesting biological functions and health-promotional effects. However, their low water solubility and stability due to sensitivity against environmental and process stresses (e.g., oxygen, light, temperature, and humidity) and low bioavailability restrict their pharmaceutical, cosmetic, and food applications [5,12]. Furthermore, essential oils, fish oil, and some other nutraceuticals have special unpleasant flavors and aromas, which limit their direct addition into food and nutraceutical formulations owing to their influence on sensorial quality [146]. The poor solubility of this type of active compounds is often further complicated if they have a short biological half-life or a site-specific absorption, e.g., only in the stomach or the upper intestine, as the transit time in these parts of the gastrointestinal tract is often variable and usually comparatively short, hence adding to the low bioavailability of these molecules [12].

These limitations can potentially be overcome by using delivery strategies, in which bioactive compounds are entrapped into carrier materials that protect them against unsuitable circumstances (during processing, storage, and digestion), allowing for the improvement of the solubility, stability, bioavailability, and biological activity of bioactive compounds, as well as their controlled and targeted release [146,147]. On the other hand, periodic administration of an active compound by oral ingestion results in constantly changing the systemic active concentration in the bloodstream. This often produces a sharp initial increase in concentration to a level above the recommended, followed by a fast decrease in such concentration below the minimum effective value [148]. Delivery systems with controlled release attempt to maintain compound concentrations in the effective recommended level over a certain period, thus offering several advantages over immediate release systems, including: effective activity, precise dose control, decreased number of dosages, reduction in side effects, and improvement in consumer convenience [7]. Different studies on encapsulation of liposoluble bioactive compounds have shown that by incorporating them into sophisticated carriers, promising and favorable results can be achieved, in terms of improved stability, bioavailability, gastrointestinal release profile, and biological activity [146,147].

Several carrier materials have been described for liposoluble bioactive compounds, namely natural and synthetic polymers, lipid carriers and proteins, in the form of: (i) nano- and microparticles [5,94]; (ii) capsules [15]; (iii) films [149]; (iv) foams [150]; (v) hydrogels [151]; (vi) nanosponges [152]; (vii) liposomes [153] and (viii) emulsions [154]. Synthetic polymers, such as polyethylene glycol (PEG) and poly(lactic-co-glycolic) acid (PLGA) [155,156] have been described, however, the controversial safe administration of synthetic matters demonstrated the need for other biologically safe options [5]. Proteins, including whey protein [157], caseins [158], gelatin [159], soy proteins [160], and cereal proteins [161] are biocompatible materials that have been reported for the encapsulation of liposoluble compounds. However, proteins usually tend to aggregate close to their isoelectric point and in the presence of multivalent counter-ions and are susceptible to be disrupted under physiological conditions in the gastrointestinal tract, thus preventing the successful delivery of the encapsulated compound [162]. Lipid-based carriers i.e., liposomal vehicles [153], emulsions [154], niosomes [163], nano-structured lipid carriers (NLCs) [164,165], and solid lipid nano-particles (SLNs) [166,167,168], are safe and promising carriers to be used as potent platforms for the delivery of liposoluble compounds. Comprehensive reviews on these systems were recently presented by Panigrahi et al. and Rostamabadi et al. [169,170,171]. Nonetheless, these carriers might experience some undesirable phenomena, e.g., Ostwald ripening, aggregation, oxidation, degradation, secretion of active agents, gelation, creaming, and precipitation, resulting from their physical and chemical instability. Moreover, under the gastrointestinal tract, at low pH values and in the presence of enzymes, lipid structures are vulnerable and the controlled release mechanisms may not occur. Low encapsulation efficiencies have also been associated to these systems and, allied to the limitations previously indicated, are restricting their extensive use as delivery systems for liposoluble compounds [146,170,172]. Considerable attention has been drawn to natural polysaccharides, namely chitosan [33], cyclodextrins [34], amylose [35], alginate [36], starch [37], pectin [38] and cellulose [5,39], which are abundant, low cost, non-toxic, biocompatible and biodegradable. Furthermore, these may have the ability to bind to specific sites, enable targeted release, have site-specific enzymatic degradation, environmental triggering, and mucosal adhesion and transport [39,173,174]. From an industrial perspective, the availability and reasonable price of encapsulating agents have a great impact on choosing encapsulating materials [6].

## 4. Cellulose Systems for Encapsulation and Controlled Release of Liposoluble Compounds

**Figure 5:**
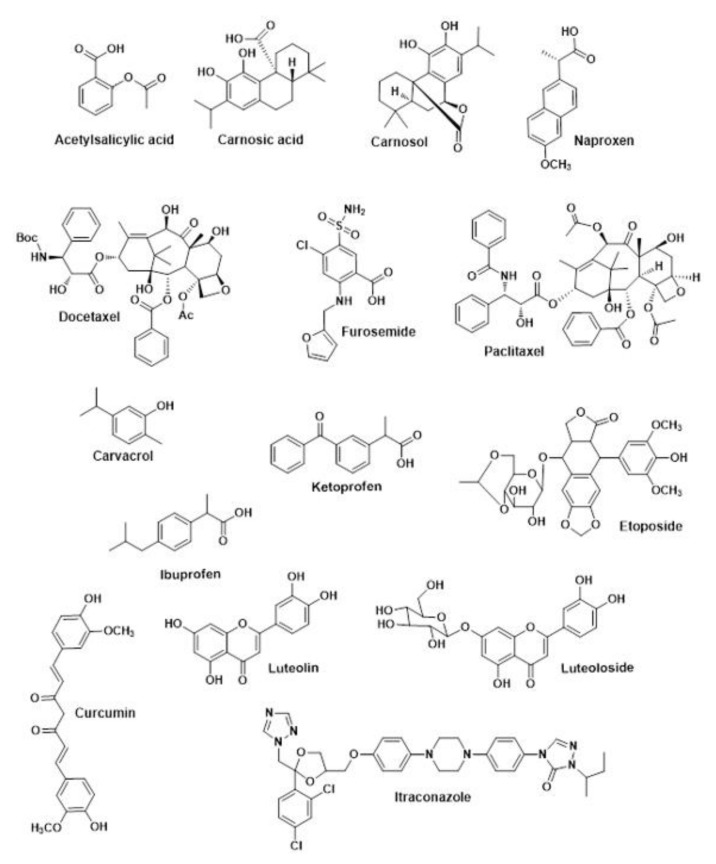
Molecular structures [114,175,176] of model liposoluble compounds that have been used as encapsulated bioactive molecules in studies using MCC, CNC, and CNF as carriers for the delivery of liposoluble compounds.

Interest in micro- (MCC) and nanocellulose (CNC and CNF) as carriers for the delivery of formulations has increased in the past few years due to their distinctive physicochemical properties, biocompatibility and biodegradability. The amphiphilic nature allied to the large surface area of these materials potentiate the adsorption of hydrophobic molecules. Previous studies have confirmed the presence of molecular interactions (such as electrostatic and hydrophobic interactions) between nanocellulose and liposoluble compounds [7,15,36], however, these will depend on the physicochemical properties of the cellulose material used [12]. Model liposoluble compounds have been used as encapsulated bioactive molecules in these studies, which molecular structures are presented in Figure 5. Amongst these, special interest has been given to curcumin, a natural phenolic compound, but also to ibuprofen, itraconazole, and paclitaxel, relevant drugs in the pharmaceutical industry. The prospect of using MCC, CNC, and CNF as vehicles for the delivery of liposoluble compounds has been discussed in relevant scientific journals for a while and will be reviewed in this section.

### 4.1. Microcrystalline Cellulose

The use of MCC in delivery formulations for liposoluble compounds is discussed in this section (Table 2). However, these uses do not involve direct molecular level control of release via binding interactions with the bioactive compound. Although the surface of MCC has a slight negative charge due to hydroxyl groups, this charge is confined to a relatively small surface area on a large mass of insoluble MCC, and would not likely adsorb or bind significant amounts of bioactives [18]. Therefore, aiming to improve its encapsulating and delivery properties, MCC is usually conjugated with lipidic systems [91,92,93], other polymers [94], or even chemically modified [83].

In a study conducted by Uesu et al., prepared and commercial MCC samples were treated with organic solvents (chloroform, acetone, ethanol, and ethyl ether) that prompted structural modifications and were used as acetylsalicylic acid (ASA) carriers [83]. The release performance was evaluated in a buffer solution at pH 4.5, and in pure water at 37 °C, conditions chosen according to the United States Pharmacopeia XXIII [177]. This states that the ordinary release of medicaments must achieve an 80% release of the active principle over 30 min in acetate buffer solution at pH 4.5, while for medicaments of controlled release, the dissolution test must be realized in a pure aqueous medium where 70% of the active principle must be released over 8 h. The ASA release occurred at a higher rate in the buffer solution at pH 4.5 than in the pure water medium. Systems containing prepared MCC were not suitable for the ordinary medicaments liberation (20–30% released after 30 min), as opposed to acetone treated commercial MCC (90% released in 30 min). Non-treated and chloroform treated prepared MCC samples were suitable for controlled release (80% released in 8 h), while for commercial MCC only ethyl ether treated samples were not suitable for medicaments of controlled release (45% released after 8 h). The ASA release from the systems is therefore sensible to structural cellulose modifications. The authors concluded that it is possible to obtain a large range of ASA release rates from MCC/ASA tablets, attaining liberation rates of controlled release and normal liberation medicaments for pharmaceutical applications, depending on surface chemistry and source of the MCC used.

**Figure 6:**
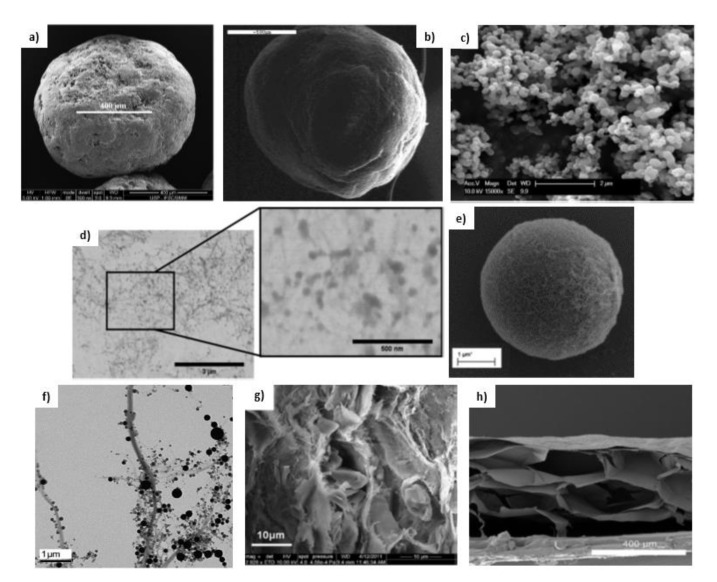
SEM images of: (**a**) MCC microparticles coated with a lipid-based system loaded with Rosmarinus officinalis extract added with maltodextrin and gum arabic [91]; (**b**) liquid-pellets of naproxen (as model compound) containing MCC, Tween 80, hydrophilic fumed silica and sodium starch glycolate [93]; (**c**) MCC particles coated with curcumin (as model compound) and PVP [94]; (**d**) nanocomplexes of tannic acid and decylamine modified CNC encapsulating curcumin [5]; (**e**) CNC and chitosan microcapsules encapsulating curcumin [15]; (**f**) HFBI coated nanoparticles entrapping itraconazole immobilized in a CNF and trehalose matrix [144]; (**g**) itraconazole loaded CNF films [149]; (**h**) CNF nanofoams loaded furosemide [117]. Reprinted with permission from refs. [5,15,24,25,122,124,150,176]. Copyright 2020 Elsevier.

Benelli and Oliveira developed MCC particles spray-coated with a lipid-based system loading *Rosmarinus officinalis* extract (including the liposoluble carnosic acid and carnosol) [91]. These MCC were mixed with maltodextrin and either with gum arabic or whey protein concentrate by a fluidized bed process. Spherical granules with good flow properties and a size of 600–800 µm by SEM analysis (Figure 6a) were obtained, exhibiting coating efficiencies of 65–80% and encapsulation efficiencies (EE) of 80–90% for both carnosic acid and carnosol. The authors concluded that the MCC retained high contents of bioactive compounds, i.e., showing potential to be used as a phytopharmaceutical active ingredient in nutraceutical, cosmeceutical, and/or veterinary formulations. Khan et al. developed paclitaxel loaded protransfersome powder formulations using a phospholipid (soya phosphatidylcholine), cholesterol, a carbohydrate carrier (MCC, lactose monohydrate or starch), and a surfactant (Span 80, Span 20 or Tween 80) via a slurry-based method, followed by compression into novel paclitaxel-loaded protransfersome tablets [92]. The MCC systems were successful in paclitaxel encapsulation, exhibiting EE of 92–98%, depending on the formulation. These systems also presented good flow properties, with flowability increasing as the carbohydrate ratio increased.

In a study by Lam et al., liqui-pellets of naproxen containing MCC, Tween 80, hydrophilic fumed silica, and sodium starch glycolate were extruded and spheronised [93]. This system showed a spheroid morphology by SEM analysis with ca. 1 mm diameter (Figure 6b), and excellent flow properties, including liqui-pellets with a high liquid load factor of 1.52. Naproxen release profile was tested in HCl buffer solution at pH 1.2 or in PBS solution at pH 7.4 to simulate gastric fluid or intestinal fluid, respectively. The formulation optimization allowed the release of 100% naproxen in 15 min at pH 7.4, while the non-optimized pellet only achieved an 80% release after 2 h. At pH 1.2, the optimized system had ca. 10% more release percentage than the non-optimized formulation after 2 h. It was found that the improved release rate was due to an enhanced disintegration of the MCC-based pellet, which when the water content is reduced during liqui-pellet production is capable of fast and even explosive disintegration, rendering it a potentially commercially feasible delivery system.

In a study by Matos et al., curcumin and poly-(vinyl-pyrrolidone) (PVP) were co-precipitated and simultaneously coated onto the surface of MCC particles through a single step association of supercritical anti-solvent and fluidized bed processes [94]. A free-flowing powder was obtained and it showed a spherical and uniform morphology with 140 µm diameter by SEM analysis (Figure 6c). Complete dissolution of curcumin into a sodium dodecyl sulfate solvent (a common model surfactant approved for oral formulations) was achieved in the first 5 min of the test. In the same time interval, raw curcumin dissolved only 3%, while curcumin/PVP/MCC particles dissolved 50% after 1 h. The improved dissolution properties of the co-precipitates were attributed to the dispersion of curcumin within the PVP matrix, which lead to the formation of amorphous particles (confirmed by DSC) and consequent improvement in the dissolution rate of curcumin, while the size of the host MCC particles contributed to superior flow properties. The authors concluded that this system has a great potential for applications in the pharmaceutical field, but also in other powder processing industries in which the release of bioactive compounds is needed.

As mentioned previously, MCC does not adsorb or bind significant amounts of liposoluble compounds, which does not make it an ideal encapsulating material on its own. However, when MCC was combined with lipidic systems, namely with a phospholipid, a surfactant, and cholesterol [92], EE superior to 90% was obtained. Furthermore, MCC is not an excellent delivery material, due to its low porosity and low bulk density, which make it practically non-disintegrating. However, the design of systems containing other polymers, such as sodium starch glycolate and PVP led to superior dissolution properties of the bioactive compounds [94,95]. The use of MCC in delivery systems is advantageous when superior flow properties are needed [91,93,94] and specific formulations combining MCC, lipid systems, and other polymers have great potential for several applications (e.g., pharmaceutical, food, and cosmetics).

### 4.2. Cellulose Nanocrystals

Nanocellulose, namely CNC and CNF, has been recently investigated as a delivery material for liposoluble compounds. In contrast to MCC, its high surface facilitates a high level of bioactive compound bound to its surface, thus providing a high loading capacity, superior encapsulation efficiency, and optimal control of dosage. Nanocellulose is predominantly hydrophilic, binding significant quantities of hydrosoluble compounds, but its surface also shows an ability to bind hydrophobic biomolecules [5,174]. Its physicochemical properties also allow nanocellulose to stabilize oil/water and air/water interfaces [18]. Nevertheless, surface modification or coupling with other materials can be necessary to modulate and optimize the loading and release of some bioactive compounds [5]. CNC for the delivery of liposoluble compounds have been structured in different forms, such as: (i) nanoparticles or nanocomplexes—matrix structures that encapsulate the bioactive compounds within the submicron-sized solid particles or absorb them at their surface [28,39]; (ii) microcapsules—which have a vesicular structure with a central core enclosed by a polymeric membrane and the bioactive compounds may dissolve into the inner core or adsorb onto the capsule surface [15]; (iii) films—thin layers of material spanning from a nanometer to several micrometers in thickness [15,178], and (iv) hydrogels—networks of hydrophilic polymer chains with an open and porous structure that can carry and release compounds in a controlled manner (the swelling of the polymer chains leads to enlargement of pores that facilitate compound release into the dissolution medium) [179,180,181]. In this review, the use of CNC in delivery formulations for liposoluble compounds over the past decade is summarized in Table 3 and the most relevant studies are highlighted in this section.

Typically, cationic and hydrophobic modifications or coupling with cationic polymers have been done in order to use CNC as a carrier for liposoluble compounds. The first study on using CNC as delivery material was reported by Jackson et al., where CNC modified with the cationic surfactant cetyltrimethylammonium bromide (CTAB) was used for the encapsulation of docetaxel, paclitaxel, and etoposide [18]. At the highest CTAB concentration (12.9 mM) the EE of docetaxel and paclitaxel was ca. 90%, while the EE of etoposide was 48%. After an initial burst release (ca. 20% in 1 h), these compounds were released in a controlled manner over 2 d (40–75%) into PBS (pH = 7.4), establishing the potential of CNC as a delivery material in pharmaceutical applications. This CNC modified material was used to encapsulate luteolin and luteoloside, and controlled release studies into PBS also showed a sustained release (45–72%) over 1 d [114]. In a study performed by Zainuddin et al., CTBA modified CNC was further used to encapsulate curcumin [39]. The prepared CTAB–CNC nanoparticles were able to bind a significant level of curcumin (i.e., EE of 80–96%), while unmodified CNC only demonstrated an EE of 27%. developed tannic acid and decylamine modified CNC (Figure 6d) for the encapsulation of curcumin, as a substitute of CTAB surfactant, which might interact with the phospholipid bilayers of cells and lead to cell death (as studied for fibroblasts cells) [5,182]. The modification achieved a remarkable EE of curcumin (95–99%) in comparison with the unmodified CNC (8–54%).

Combining CNC with chitosan has also been a commonly adopted system to encapsulate liposoluble compounds. Mohanta et al. employed a layer-by-layer approach for the fabrication of CNC and chitosan multilayer films and microcapsules for the encapsulation of curcumin [15]. In the case of microcapsules (Figure 6e), curcumin was incorporated into the wall of the capsules, leaving the aqueous core available for further loading of a hydrophilic compound, enabling dual bioactive compound delivery. After an initial rapid release of curcumin (35% in 1 h), it was released in a sustained manner (65% over 8 h) into PBS buffer (pH 7.4). This study also showed that the binding of curcumin with CNC was through hydrogen bounding and Van der Waals interactions, and theoretically modeled the interaction of other lipophilic compounds with CNC by molecular docking. These compounds showed binding energies comparable to that of curcumin, which anticipated that CNC can also be used as a carrier for other lipophilic compounds.

De Castro et al. developed TEMPO-oxidized-CNC (TOCNC) films modified with hydroxypropyl-β-cyclodextrin (HPβCD) to encapsulate carvacrol and curcumin [178]. The loading of carvacrol and curcumin into HPβCD -grafted TOCNC increased by forming inclusion complexes when compared with the virgin TOCNC. The presence of HPβCD also induced a slowdown in the release of both carvacrol and curcumin into distilled water, highlighting these structures potential application as antibacterial products in food packaging, as well as in other fields.

Systems with alginate [29], PLGA [183], gelatin and collagen [184] have also been developed for the encapsulation and release of curcumin and ibuprofen. Nonetheless, modification with CTBA has allowed a significant improvement in the encapsulation of liposoluble bioactive compounds with EEs of ca. 90% [18,39], while coupling with chitosan has enabled EE of 70–80% [28]. Typical release profiles are characterized by a biphasic trend with a fast initial burst release in the first few hours (0.5–2 h) followed by a slower release phase. CTBA modification enabled a prolonged release of bioactive compounds of 45–75% over 1–2 d [18,114], while coupling with chitosan achieved a controlled release of 40–65% over a shorter period (i.e., 6–12 h) [15,28,179,180]. When release studies were performed into simulated gastric or acidic conditions (using chitosan as a copolymer), a faster release of curcumin could be observed [170,180].

### 4.3. Cellulose Nanofibers

Similar to CNC, CNF for the delivery of liposoluble compounds have been structured in different forms. These include: (i) microparticles, usually obtained by spray drying a CNF suspension containing the bioactive compound [115,118]; (ii) microcapsules [185,186]; (iii) films [149] and (iv) foams—macroporous materials fabricated from hydrogels in which the liquid has been replaced by gas (air) [150]. In this review, the use of CNF in delivery formulations for liposoluble compounds over the past decade is summarized Table 4 and the most relevant studies are discussed below.

The first study on using CNF as a delivery material for liposoluble compounds was reported by Valo et al., where protein-coated nanoparticles entrapping itraconazole were immobilized in a CNF and trehalose matrix (Figure 6f) [144]. The results demonstrated that CNF played a critical effect on particles stabilization and prevented their aggregation during freeze-drying and storage. Release studies into a NaCl/HCl solution (pH 1.2) (simulating the gastric fluid) showed an initial burst release of itraconazole (ca. 60% in 10 min), followed by a slower release (90% in 90 min), which was generally maintained after 12 weeks of storage (75% release in 90 min). The resulting highly porous nanoparticle formulation showed an increased in vitro and in vivo performance compared to plain itraconazole. Kolakovic et al. prepared itraconazole loaded CNF films with unmodified CNF by a filtration method (Figure 6g) [149]. Loading capacities of 20–40% and EE > 80% were achieved. The films, exhibiting excellent mechanical properties, were suitable for the incorporation of heat-sensitive compounds. Release studies using a NaCl/HCl solution (pH 1.2) showed a long-lasting (up to 3 months) sustained release, which was believed to be due to the tight nanocellulose network formed around the itraconazole crystalline entities. This film structure of unmodified CNF achieved a far more prolonged release than the previous system by Valo et al. [144]. Due to their long-lasting release, such systems were reported to be less feasible for oral delivery, however, they may be useful in implants, transdermal patches, or ocular applications.

CNF systems using only unmodified CNF have also been proposed for the encapsulation of ibuprofen and furosemide. Kolakovicet et al. produced CNF microparticles containing ibuprofen by a spray drying method [118]. Release studies showed a long-lasting sustained release profile over 2 months into PBS (pH 7.4), proving that unmodified CNF can sustain release by forming a tight fiber network, thus limiting compound diffusion from the system. Svagan et al. developed dry foams consisting of CNF and the model drug furosemide at loadings of 21% and 50% (w/w) by simply foaming a CNF suspension followed by drying [117]. Compared to a marketed tablet formulation, which disintegrated within a couple of minutes, the flexible and porous foams (Figure 6h) showed a controlled release (45–65% over 24 h) in simulated gastric fluid (pH 1.6). The authors suggested that the floating CNF foam could potentially be used as a gastric retentive system since furosemide has a very site-specific absorption in the stomach and upper intestine. This foam system, however, showed a faster release of furosemide, when compared to the long-lasting (2–3 months) sustained release observed in the previously discussed studies for ibuprofen (micropareuropeticles) and itraconazole (films) [118,149].

Systems combining CNF with other materials, such as gum arabic and lipids, have also been proposed in the literature for the delivery of liposoluble compounds [143,175]. Although these studies seem promising and present innovative systems, EE and release studies have not yet been explored.

It has been shown that CNF systems using only unmodified CNF can control and sustain the release of liposoluble bioactive compounds. Both long-lasting (2–3 months) and shorter (24 h) release profiles can be obtained, depending on the encapsulated bioactive compound, the designed structure (microparticles and films vs. foams), and the CNF source. Nonetheless, more complete and detailed studies, e.g., analyzing encapsulation efficiencies, would be useful for a more comprehensive comparison and discussion.

Generally, nanocelullose systems show improved performance in the controlled delivery of liposoluble compounds, when compared to microcellulose due to its small surface area, and low porosity and bulk density. To achieve superior encapsulating and delivery properties, MCC was conjugated with lipidic systems and other polymers, such as sodium starch glycolate and PVP. Nanocellulose on the other hand, especially CNF, can be used on its own to control and sustain the release of liposoluble compounds, by forming a tight fiber network around the bioactive compound to be released. In the case of CNC, cationic or hydrophobic surface modification, or coupling with cationic polymers, can be necessary to modulate and optimize the loading and release of specific bioactive compounds, where CTBA modification has been the most successful. MCC however is advantageous when superior flow properties are needed. Even though rod-shaped nanoparticles have been good carriers due to long circulatory time and high cellular uptake in the body, they show a high tendency to aggregate, poor flowability, and difficult handling. To overcome these drawbacks, nanocellulose delivery systems have been structured into different forms (e.g., microparticles, capsules, films, hydrogels, and foams). Other crucial factors for the development of successful micro or nanocellulose delivery systems include: (i) the selection of the encapsulation method, (ii) carrier-bioactive interactions, and (iii) the different sources and extraction methods of the cellulose materials, which may render different release capabilities.

## 5. Safety and Potential Toxicity of Cellulose Micro and Nanostructures

In order to propose the safe use of cellulose-based micro and nanostructures for food, cosmetic and pharmaceutical applications, it is important to evaluate the toxicity and cellular uptake of these materials. Regarding cellulose microstructures, minimal oral, dermal, and inhalation toxicity, as well as non-irritating and non-sensitizing effects to the skin have been reported [90]. Evaluation of overexposure’s chronic effects has reported MCC as inert dust and nontoxic to the lungs, as well as non-genotoxic. For example, Kotkoskie et al. conducted a subchronic toxicity study to evaluate the potential effects associated with MCC [187]. The ‘no observed adverse effect level’ (NOAEL) for toxicological effects was greater than 5000 mg/kg/day, the highest MCC dosage tested. In a report by the World Health Organization [188], the committee concluded that the existing toxicological data provided no evidence that MCC could cause toxic effects in humans when used according to good manufacturing practices, verifying its safety in food and pharmaceutical applications. In a recent report on the re-evaluation of MCC as a food additive following a request from the European Commission, the panel concluded that there were no safety concerns at the reported applications and use levels for the unmodified and modified MCCs. The determined acute toxicity of MCCs revealed to be low and without genotoxic concern. Short-term and subchronic dietary toxicity evaluation did not reveal adverse effects. Chronic toxicity studies showed NOAEL values up to 9000 mg/kg/day, no carcinogenic properties or adverse effects on reproductive performance were observed [189].

Regarding the use of nanostructures in the encapsulation of bioactive compounds, there are potential limitations in terms of its nano-dimensions, which can allow these materials to pass through physiological barriers after oral consumption, inhalation, or skin permeation [13]. This can increase the absorption and bioavailability of encapsulated compounds and may enhance health outcomes, but can also result in cell toxicity due to the absorption of the carrier materials. In 2011, the European Food Safety Authority (EFSA) put forth guidelines for risk assessment of the incorporated nanomaterials within food matrixes. Until now, in vivo and in vitro tests have been conducted to observe the effects of food, cosmetic and pharmaceutical nanostructures, but further studies are needed to extend their global commercialization [169].

Toxicology studies of cellulose nanomaterials, comprehensively reviewed by Roman and Endes et al., are still in an early stage and mainly focus on cytotoxicity [190,191]. Overall, studies have shown low-to-minimal adverse health effects from oral or dermal studies, but pulmonary and cytotoxicity (i.e., effects on cell viability) studies have yielded conflicting results [52]. The cytotoxicity of CNCs against nine cell lines: murine macrophage cells (RAW 264.7), human brain microvascular endothelial cells (HBMEC), mouse brain endothelial cells (bEnd.3), human mammary epithelial cells (MCF-10A), human breast cancer cells (MDA-MB-231 and MDA-MB-468), human prostate cancer cells (PC-3) and glial cells (C6) has been studied. Cytotoxicity was evaluated upon both cellular metabolism (MTT assay) and cell membrane integrity (LDH assay), and no cytotoxic effects of CNC in the concentration range and exposure time studied (0–0.05 mg/mL for 48 h) were reported [192]. Tests with fibroblasts (L929) showed that dispersions of CNCs with concentrations in the range of 0.1–2.0 mg/mL exhibited low toxicity [193]. In another study, where colon carcinoma (HCT116) and murine embryo fibroblast (NIH3T3) cell lines were confronted with CNCs by WST-1 assay, the results revealed that CNCs did not present any substantial cytotoxicity at the various concentrations tested (0.01–0.25 mg/mL) [194]. Regarding CNF, no inflammatory effects or cytotoxicity on mouse and human macrophages have been reported [195]. In a recent study conducted by Lopes et al., Alamar Blue and LDH assays showed that CNF does not have adverse effects on the metabolic activity and membrane integrity of immune (THP-1 macrophages), dermal (HDF), and lung (MRC-5) cells [196]. Furthermore, no significant reactive oxygen species production by THP-1 macrophages was found, suggesting that the oxidative potential of the cells was not affected, and no cellular uptake was observed by TEM.

Deloid et al. evaluated the toxicological effects of ingested CNC and CNF in in vitro intestinal epithelium (triculture model—2 Caco-2, HT-29MTX, and Raji B cells; 24 h incubation) and in vivo rat models [197]. No cytotoxicity (LDH assay) or increase in reactive oxygen species was observed in vitro, nor significant differences in hematology, serum markers or histology between controls and rats which were given NC suspensions.

Nevertheless, some toxicity studies on nanocellulose materials have reported time- and dose-dependent effects. Burchett investigated the toxic effects of CNCs on eukaryotic organisms (*Saccharomyces cerevisiae*) and human embryonic kidney (HEK-293) cells, using an auto-bioluminescent method, and reported that at 1 mg/mL CNCs led to a decrease (60%) in metabolic activity of HEK-293 after 48 h [198]. No significant changes in metabolic activity were observed for a concentration between 0.001 and 0.01 mg/mL for the same period of exposure. Pereira et al. evaluated the in vitro cytotoxicity and the effect on gene expression of CNF in bovine fibroblasts cells and reported that low concentrations (0.1 mg/mL) had no cytotoxicity, whereas high concentrations (2.0–5.0 mg/mL) caused a sharp decrease in cell viability and affected the expression of stress- and apoptosis-associated molecular markers [199]. However, it is important to notice that these concentrations are much higher than those expected to be used in food and pharmaceutical applications. In addition to dose and time, other properties have been reported to influence the toxicity of cellulose nanomaterials, including size, morphology, crystallinity, and surface chemistry, as these properties play a vital role in cell–biomaterial interactions [13]. Mahmoud et al. explored the effect of surface charge of CNCs on cellular uptake and cytotoxicity [200]. Negatively charged fluorescein isothiocyanate (FITC)-labeled CNCs was evaluated and compared against the positively charged rhodamine B isothiocyanate (RBITC) labeled CNCs in HEK 293 and insect *Spodoptera frugiperda* (Sf9) cells. The in vitro cellular uptake studies showed that the positively charged CNC–RBITC was uptaken by the cells without any noticeable cytotoxic effect, with no significant internalization of negatively charged CNC–FITC being observed at physiological pH, however, the cells were surrounded by CNC–FITC, leading to eventual cell rupture. In another study, CNF were surface-functionalized with anionic and cationic groups, and the effect on monocyte/macrophage (MM) reaction was investigated along with the unmodified form. A pro-inflammatory phenotype was found to be activated by the anionic carboxymethylated NFC films, while the unmodified forms promoted a mild activation and cationic hydroxypropyl-trimethylammonium groups did not cause the activation of MMs [201]. Interestingly, in a study by Alexandrescu et al., in comparison with no acute toxic phenomena for unmodified CNF, modified-CNF with cross-linking agent polyethyleneimine and cationic surfactant CTAB caused a significant reduction in cell (fibroblasts 3T3) viability and proliferation [182]. Cellulose nanostructures may present a random distribution of surface charges as well as complex stereochemical behavior and poly-disperse size distribution, which make it difficult to compare different toxicological studies. Male et al. investigated the impact of different CNCs sources (hemp, flax, and cellulose powder) on the cytotoxicity of two different cell lines: Chinese hamster lung fibroblast (V79) and Sf9 insect cells [202]. The authors observed that flax exerted the highest cell growth inhibition on Sf9 cells compared to cellulose powder and hemp, but CNCs did not exhibit significant cytotoxicity in the studied cell lines. They suggested that a correlation between the inhibitory effect and the carboxylic acid content of the CNCs exists. Hosseinidoust et al. also evaluated the carboxyl content of CNCs [203]. Interactions with different tissue cell lines (colon epithelium Caco-2, cervix epithelium HeLa, kidney epithelium MDCK, and macrophage J774) were evaluated and the uptake of CNCs by these cells did not show prominent damage or changes in cell density on the membrane, but at higher carboxyl contents (over 3.9 mmol/g), a charge-dependent decrease in mitochondrial activity was observed. On the other hand, in a study by Liebert et al., confocal micrographs of human foreskin fibroblasts after 48 h of incubation with cellulose nanoparticles exhibiting spherical morphology indicated high CNC uptake into cells [204]. In contrast to normal CNCs, rapid cellular uptake was found for the spherical CNC, which indicated the influence of morphology on endocytosis.

Although there is no clear evidence of a serious influence or damage of cellulose nanomaterials on the cellular and genetic level in applied concentrations, the inhalation of plentiful nanocellulose (especially for CNC) may induce pulmonary inflammation due to self-aggregation and bioaccumulation of the cellulose material in the body [72,205,206,207]. However, it is important to note that direct inhalation of CNC powder is not likely when consuming cellulose nanomaterials incorporated into formulations since these materials are normally structured into systems (e.g., microparticles, hydrogels, films) which have been classified as non-toxic in existing studies [208,209].

The possible toxic effects of nanocellulose in vivo were evaluated in several studies. Acute oral toxicity was evaluated by oral administration to rats (doses of up to 2000 mg/kg) without observation of adverse effects of CNCs. Guinea pigs and mice were also exposed to CNCs by intradermal injection and topical application and the results showed non-sensitizing effects at the tested concentrations. Concerns over eco-toxicological risks associated with nanocellulose have also arisen and some studies on this topic have been performed. CNCs were tested to a broad eco-toxicological panel through toxicity assays, including rainbow trout hepatocytes and nine aquatic species, such as *Ceriodaphnia dubia, Daphnia magna*, rainbow trout (*Oncorhynchus mykiss*), and fathead minnow (*Pimephales promelas*). CNCs were found to have low toxic potential and environmental risk, showing no genotoxicity and no effects on survival and growth of aquatic organisms at concentrations that could occur in receiving waters [210]. The biodegradability of CNCs in an aqueous environment was also studied as per the Organization for Economic Cooperation and Development (OECD) standard and compared with other nanomaterials. CNCs nanoparticles were found to biodegrade at similar levels but faster than materials such as fullerenes and carbon nanotubes, demonstrating potential environmental advantages over these nanomaterials [52,211].

Overall, the studies conducted so far reported the absence of serious environmental and biological concerns. However, a few studies demonstrated that cellulose nanomaterials might cause some toxic effects and more studies are necessary to better clarify this issue. Studies investigating more deeply the in vitro and in vivo cell interactions, the effects, and mechanisms of aggregation in the body, the possible side effects upon ingestion or skin contact, the acute and/or chronic toxicity (at normal conditions and with pre-existing disease conditions), as well as the bio-distribution and fate of these materials, are needed. It is also necessary to assess the potential risks associated with modified cellulose nanomaterials since small chemical modifications of the material surface could result in distinct toxicity profiles. In sum, future investigations are needed to comprehensively characterize the toxicology of different types of cellulose nanomaterials, both in vitro and in vivo, which would provide consistent and useful knowledge that can guide the outgrowth of regulatory norms and guidelines. Due to increasing interest in the application of cellulose-based micro and nanomaterials, their potential toxicity is an issue of utmost importance that deserves special attention and investigation.

## 6. Digestibility, Bioaccessibility, and Bioavailability

Oral delivery is the preferred route for bioactive compound administration over other routes, as it is non-invasive, cost-effective, and the easiest and most convenient method for compound delivery [212]. The understanding of solubility, digestion, absorption, and metabolism of bioactive compounds is key before the design of a delivery system to overcome their bioavailability limitations [213]. A bioactive compound can only be effective once it has dissolved and permeated through the intestinal barrier, where the absorption of most molecules occurs. Bioaccessibility has been defined as the fraction of a compound that is released from a matrix in the gastrointestinal (GI) lumen and thereby made available for intestinal absorption. Bioavailability is generally defined as the fraction of a compound that can be absorbed and becomes available at the site of action for physiological functions and/or storage [214,215].

For liposoluble compounds, the digestion starts with its release from the formulation. If lipids are present, liposoluble compounds are dissolved in the fat phase of the matrix, followed by partial gastric hydrolysis, emulsification into lipid droplets of gastric emulsion, and further lipolysis by pancreatic lipases. They are then transferred into mixed micelles, composed of by-products of lipid hydrolysis (mainly free fatty acids and monoacylglycerols), phospholipids, cholesterol, and bile salts. The fraction of liposoluble compounds incorporated into the mixed micelles is bioaccessible, while the concentration of liposoluble compounds in the plasma is bioavailable [213]. Due to the limited solubility of liposoluble compounds in aqueous solutions, including saliva and GI fluids, allied to their low dissolution rate, and high metabolism in the GI tract, these compounds usually exhibit low bioaccessibility and bioavailability [214,216]. Furthermore, owing to the physiology of the small intestine, with the presence of a water layer across the intestinal barrier, the absorption of liposoluble compounds can be compromised [217]. Absorption occurs when liposoluble compounds are taken up by enterocytes in the small intestine, either via lipid transporters or by passive diffusion, being metabolized and secreted as chylomicrons (lipoprotein globules that transport dietary lipids) into the lymphatic system [213,215]. Although these compounds can be highly permeable, the poor solubility results in a low concentration gradient between the gut and the blood vessels, limiting their transport and absorption [218]. Thus, the solubilization, dissolution rate, absorption by intestinal epithelium cells, and transformation within the GI tract are key factors impacting bioaccessibility and bioavailability [213,214].

To evaluate bioavailability and bioaccessibility, in vitro methods such as solubility, dialyzability, and gastrointestinal digestion (chemical and enzymatic simulation, cell culture, and colonic fermentation models), as well as in vivo studies can be performed, allowing the determination of plasma exposure represented by AUC (area under the plasma drug concentration-time curve) [219,220]. In vitro digestion methods using cells mimicking the human intestinal epithelium (e.g., Caco-2 cell line, HT29 cell line, and co-culture models) are convenient, reproducible, and cost-effective. They give a good estimation of the amount of the bioactive compound available for uptake, although they possess some limitations, such as lack of mechanical forces and gastric emptying, and absence of host response factors. In contrast, ethical considerations associated with human volunteers and high costs are potential barriers to in vivo studies [213,214]. The strengths and drawbacks of these approaches were discussed by Etcheverry et al. and Kamiloglu and Capanoglu [219,220].

As a result of their low bioaccessibility and bioavailability, losses of liposoluble compounds between 8 to 40% after in vitro digestion [221,222] and between 75 to 96% after colonic fermentation were generally observed [223]. A study by Garcea et al., which investigated levels of curcumin and its metabolites following oral administration (450–3600 mg of curcumin), provided the first evidence in humans that oral administration of curcumin furnishes trace levels of the parent compound and its metabolites in the liver and portal circulation [224]. The lack of quantifiable levels of curcumin in plasma was consistent with clinical reports in which doses of up to 180 mg of curcumin failed to establish detectable plasma levels and very high doses (up to 8 g) yielded curcumin peak levels of only ca. 0.5–2 μM within 1 h of oral administration [225].

**Figure 7:**
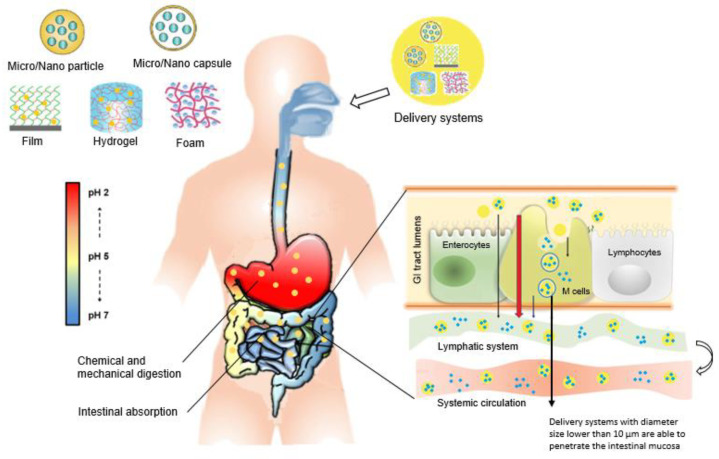
Schematic overview of the gastrointestinal fate of micro and nano delivery systems Adapted with permission [234,235], 2020, Elsevier.

In order to preserve their properties and improve their bioavailability, an effective approach is represented by using functional delivery systems, which should be able to solubilize/disperse liposoluble compounds in the gastric and intestinal contents after oral administration, leading to enhanced bioactivity in the final product (Figure 7) [147,213,226]. Entrapment of liposoluble compounds into biopolymeric micro- and nanosystems can improve stability against degradation in GI conditions, enhance solubilization and dissolution and increase the resident time in the GI tract, and promote the transfer to enterocytes allowing for targeted GI delivery, thus enhancing bioavailability and bioaccessibility of these compounds [227,228]. Formulations such as tablets exhibit a low surface-area-to-volume ratio, limiting the rate of diffusion of the bioactive compound. In addition, the compound release is confined to a small area of the GI tract, causing high local concentrations [229]. Micro- and nanosystems have a large surface-area-to-volume ratio, which offers a larger interface for partitioning and release. Delivery systems with diameter sizes lower than 10 μm may enter the intestinal mucosa, allowing for longer residence times in the intestine and closer contact between the dosage form and the site of absorption [229,230]. Nanosystems have a higher uptake by intestinal epithelia compared to micro counterparts as a result of reduced size and surface properties, which makes nanosystems able to penetrate through the cell wall and approach the target cells, releasing their contents properly [146]. By improving the surface-area-to-volume ratio micro- and nanosystems raise mucoadhesive prospects in the small intestine, by increasing the viability of intermingling with enzymes or metabolic factors [147]. Furthermore, these stimulate the release of pancreatic and bile juice, which may assist the digestion of the carrier material and enhance compound absorption [170]. Poorly soluble compounds within multiparticle formulations have shown major advantages in bioavailability when compared to bulk compound materials [144,145,231], as these distribute more uniformly in the GI tract, resulting in a more uniform release and a reduced risk of local irritation [229].

Micro- and nanocellulose structures have been used as delivery systems to improve the bioavailability of liposoluble compounds. Madhavi and Kagan developed a micronized formulation consisted of lipids, hydroxypropyl methylcellulose, and sodium alginate as a base matrix that was able to encapsulate 25% of curcumin allowing its sustained release in the intestine with the concomitant improvement of its absorption [232]. The authors showed that the microsystem was able to improve the curcumin bioavailability 9.7 times as compared to unformulated curcumin, in a single-dose bioavailability study conducted in healthy human volunteers [232]. On the other hand, Mantas et al. demonstrated that nanocellulose was able to increase the bioavailability of ibuprofen due to the high surface area of such nanostructures [233]. These authors showed that ibuprofen encapsulated in nanocellulose exhibited substantially improved pharmacokinetic parameters (i.e., AUC increased almost seven times, while mean residence time and t_1/2_ were halved) than microcellulose counterparts through a pilot pharmacokinetic in vivo study performed in rats. As discussed above, several strategies comprising the addition of cationic groups or coupling with cationic polymers aiming to improve the performance of NC as a carrier for liposoluble compounds have been investigated. Wang and Roman developed complexes synthesized from CNCs and chitosan, which have provided a long residence time in the small intestine due to the attraction between the negatively charged intestinal mucosa and positively charged chitosan, providing an efficient platform for oral drug delivery [229]. The obtained structures had a few hundred nanometers to several micrometers, allowing penetration of the intestinal mucosa, which is beneficial in oral delivery applications as it leads to longer residence times of the formulation in the small intestine and release of the compound near the epithelium, through which the compound is absorbed. On the other hand, the combination of cellulose structures with lipid systems is also desirable, as the presence of dietary lipids is mandatory for the solubilization of liposoluble compounds in the GI medium and transference to mixed micelles, since only the compounds within micelles are bioaccessible and eligible for absorption [213].

At the same time, it is of crucial importance to know the fate of ingested cellulose structures while being passed throughout the GI tract. Due to the lack of cellulases in the human small intestine, it can be concluded that cellulose-based materials are not degraded significantly following oral administration [236]. However, it is worth noting that the micro and nano-size of these structures and pH variance in different GI compartments (which may have an impact on the aggregation and surface chemistry of cellulose structures) play a role in their digestion and absorption. Although sulfated (H_2_SO_4_-generated) nanocellulose suspensions remain homogeneously suspended in physiological pH values, aggregation may occur due to desulfation by the gastric acid [237], mild alkalization in the duodenum [96], or neutralization by cations (e.g., sodium), since these avoid the inter-particle repulsive interactions [190]. The aggregated NC may display altered properties such as longer residence time within the GI tract and less effective diffusion coefficient through the mucus layer. The longer residence time within the GI tract is an opportunity for NC interaction with the gut microbiome, however, it is uncertain from the existing literature whether these interactions occur and if they are positive, negative, or inconsequential [236]. As the mucus layer of the GI tract is negatively charged, negative particles exhibit higher transport rates than neutral or positive structures, due to the electrostatic repulsion between diffusing particles and the negative membrane [238]. Sulfated NC can therefore penetrate the mucosa and deliver directly into the bloodstream, even though this may be restricted by NC desulfation/neutralization or by considerable longitudinal size, as penetration is dependent on size and surface charge [239]. Due to the small pore size (roughly 3 nm) of the vascular epithelium, endocytosis-absorbed NC is most likely transported through the lymphatic system than the venous circulation [236]. A scenario in which some amounts of NC structures are absorbed through an endocytotic mechanism, such as phagocytosis, has been considered [236]. A study on cellulose nanospheres and CNC (80–260 nm) revealed the influence of nanoparticles geometry on endocytosis. In contrast to the rod-shaped CNCs, fast cellular uptake was observed for the nanospheres [204].

The first study determining the fate of nanocellulose during GI digestion was performed by Liu et al. [240]. The results suggested that the behavior of each type of NC (CNF, CNC, and TEMPO-CNF) differed during digestion. For CNC, gelation after digestion was observed, leading to an increase in digesta viscosity. CNC formed a hydrogel network at the gastric phase, working as an emulsion stabilizer. CNF maintained its fibril entanglement and worked as an emulsion stabilizer at the gastric phase as well. TEMPO-CNF did not behave as a stabilizer, as aggregation led to de-swelling and phase separation of TEMPO-CNF gels—a decrease in digesta viscosity was observed. Regarding the influence of nanocellulose on nutrient digestion, the same study concluded that the ingestion of NC resulted in the delayed initial release of free fatty acids during digestion, which was also observed by Deloid et al. and Liu and Kong, thus indicating that NC can be advantageous in terms of increasing digesta viscosity and delaying initial lipid digestion [241,242]. A follow-up study by Liu & Kong demonstrated the effects of NC on milk digestion and mineral absorption [242]. Results showed that TEMPO-CNF and CNC reduced glucose diffusion, while CNF and TEMPO-CNF reduced the amount of free fatty acids produced during the intestinal digestion of milk fat. CNC delayed the diffusion of free amino nitrogen during intestinal digestion of milk proteins and adsorbed significant amounts of Zn, while all three types of NC adsorbed significant amounts of Fe. Results from this study suggest that NC when incorporated into oral formulations, may affect digestion and nutrient absorption. Lin et al. evaluated the mucoadhesive properties of the three types of NC (CNF, CNC, and TEMPO-CNF) in the digestive condition with in vitro (viscometric method, zeta potential evaluation) and ex vivo (flow-through method) assays [243]. Results revealed that the three types of NC had mucoadhesivity in GI conditions, with the level of adhesion depending on the type of NC, its concentration, and the GI compartment, thus showing the potential of NC as gastroretentive delivery systems.

Recent studies are now emerging showing the fate of cellulose micro- and nanostructures during gastrointestinal digestion, as well as their influence in digestion when incorporated into oral formulations, however further studies are needed to better elucidate this matter. Although several micro- and nanocellulose delivery systems have been designed for the release of liposoluble, their influence in bioavailability remains largely empirical, mainly because methods for testing dissolution and predict bioavailability are usually not adopted. Future studies are necessary to enhance the body of evidence that such formulations will not only be beneficial with respect to the stability of the bioactive compounds but can also enhance the bioactivity of these promising compounds via improving aspects of bioavailability.

## 7. Conclusions and Future Prospects

Advances in micro- and nanoengineering of cellulose, the most abundant biopolymer in the world, have demonstrated cellulose-material utilization possibilities that were thought impossible. Cellulose micro- and nanostructures, produced via mechanical, chemical, and enzymatic treatments from various sources, are a new class of cellulose-based ‘‘building blocks’’ that are inspiring advances in cellulose science, technology, and product development for the next generation of renewable and sustainable products.

Their unique physicochemical properties, allied to their biocompatibility and biodegradability, made them excellent candidates as carriers in delivery systems. Their hydrophilic and hydrophobic surfaces and the large surface area can potentially be used to bind liposoluble compounds, which can have very interesting biological activities, but which use has been restricted due to low water solubility, stability, and bioavailability. To date, micro- and nanocellulose delivery systems investigated include particles, capsules, films, hydrogels, and foams, individually, modified, or combined with other polymers and materials. Generally, nanocellulose systems show improved performance in the controlled delivery of liposoluble compounds when compared to microcellulose, but the use of MCC may be advantageous when superior flow properties are needed. CNF can be used on its own to control and sustain the release of liposoluble compounds, while for CNC, cationic or hydrophobic surface modification or coupling with cationic polymers can be necessary to modulate and optimize the loading and release of specific bioactive compounds. Although relevant progress has been achieved in the preparation, characterization, and application of cellulose micro- and nanostructures for delivery systems, there are still some questions that need to be answered. Challenges ahead of this field encompass understanding the behavior of micro- and nanocelluloses inside the body in terms of digestion, absorption, biodegradation, biodistribution, bioavailability enhancement, and long-term toxicity. Literature studies so far mainly report the safety and lack of toxicity of the various forms of micro- and nanocellulose, but further development of these materials as delivery systems will no doubt require more extensive investigation into their toxicity characteristics.

Indeed, cellulose micro- and nanostructures are truly versatile materials with respect to the delivery of liposoluble compounds. However, to fully prove their application, further research is needed aiming at better understanding its interaction with the encapsulated compounds, as well as the performance of such formulations in vitro and in vivo.

## Figures and Tables

**Table 1:** Isolation methods and material properties of NC to be applied as carrier in the delivery of liposoluble compounds.

Cellulose Source	NC Material	Isolation Method(s)	Isolation Conditions	Surface Chemistry	Material Properties	Product Yield	Reference
Bleached commercial softwood	CNC	AH + S	AH: H_2_SO_4_ 64% *w/w*; ratio: 8.75 mL/g; 45 °C/25 min S: 10 min, 60% power	Sulfate groups	CI: >90% Size: ca. 10 vs. 500 nm Film Morphology: layered, unidirectional ZP: −55 mV	-	[18]
Kenaf bast	CNC	AH	AH: H_2_SO_4_ 64% *w/w*; 45 °C/40 min	Sulfate groups	CI: 71.9% Size: 4–20 vs. 50–200 nm Aspect ratio: 13.4 Morphology: Rod-shaped TG: 180 °C, 230 °C	41%	[39]
Empty fruit bunch	CNC	AH + S	AH: H_2_SO_4_ 58% *w/w*; 45 °C/45 min S: 10 min, 60% power	Sulfate groups	CI: 77.6% Size: 13–30 vs. 150–360 nm Aspect ratio: 27 TG: 200 °C Contact angle: 45°	-	[5]
Cotton	CNC	AH	AH: H_2_SO_4_ 64% *w/w*; ratio: 20 mL/g 45 °C/45 min Water dialysis	Sulfate groups	Size: ca. 140 nm lenght ZP: −55 mV	25%	[95]
Date palm stalks	CNC	AH	AH: H_2_SO_4_ 64% *w/w*; ratio 20 mL/g 45 °C/45 min S: 6 min, 200 W	Sulfate groups	CI: 78% Size: 5–7 vs. 86–237 nm ZP: −53.8 mV		[28]
Micro-crystalline cellulose	CNC	AH	AH: H_2_SO_4_ 64% *w/w*; ratio: 12 mL/g; 45 °C/30 min	Sulfate groups	CI: 70.2% Size: ca. 50 nm wide Morphology: spheroid	-	[114]
Bleached kraft pulp (*eucalyptus)*	CNF	Defibrillation	1500 rpm; 5 passes	Hydroxyl groups	Size: 17–40 nm vs. 2–12 µm Morphology: twisted and elongated fibers; non-individual network	-	[115]
Bleached sulfite pulp (spruce)	CNF	HPH	1650 bar (chambers 400/100µm); 2 passes	Hydroxyl groups	CI: 48% Size: 4–6 nm vs. several µm DS: 0.44 mmol/g	-	[116]
Bleached sulfite pulp	CNF	HPH	1650 bar (chambers 400/100 μm); 2 passes	Hydroxyl groups	Size: 3–5 nm vs. several μm	-	[117]

Legend: NC—Nanocellulose; CNC—Cellulose nanocrystals; CNF—Cellulose nanofibers; AH—Acid hydrolysis; S—Sonication; HPH—High pressure homogenization; CI—Crystallinity index; x vs. y—width versus length; ZP- Zeta potential.

**Table 2:** Studies on the use of microcrystalline cellulose for the encapsulation and release of liposoluble compounds.

Encapsulating Material(s)	Active Ingredient(s)	Encapsulation Method	Final Structure	Encapsulation Results	Release Results	Application	Reference
MCC treated with ethanol, acetone, chloroform, or ethyl ether	Acetylsalicylic acid	Solvent Evaporation	Microparticles	Size: ca. 100 µm Morphology: elongated TG: 160 °C	Commercial MCC; Solvent: Buffer pH 4.5 Acetone: 90%/30 min Other solvents: 20–30%/30 min Commercial MCC; Solvent: pure water Acetone: 100%/2 h Ether: 45%/8 h Other solvents: 80%/8 h Prepared MCC; Solvent: Buffer pH 4.5 15–30%/30 min Prepared MCC; Solvent: pure water Chloroform and no solvent: 80%/8 h Other solvents: 40–55%/8 h	Pharma	[83]
MCC + Lipid system (Poloxamer 407, stearic acid) + Maltodextrin DE10 + Gum arabic or Whey protein	*Rosmarinus officinalis* extract, including Carnosic acid and Carnosol	Fluidized Bed Spray Coating	Microparticles	EE = 80–90% Coating efficiency = 65–80% Morphol.: Spherical Size: 600–800 µm Excellent flow properties	-	Food, Cosmetic, Pharma	[91]
MCC + SPC + Cholesterol + Surfactant (Span 80, Span 20 or Tween 80)	Paclitaxel	Slurry Method	Protransfersome	EE = 92–98% Morphol.: oblong ZP: −2.52 mV	-	Pharma	[92]
MCC + Tween 80 + Hydrophilic fumed silica + Sodium starch glycolate	Naproxen	Extrusion + Spheronisation	Liqui-pellet	Size: ca. 1 mm Morphol.: spheroid Excellent flow properties	Solvent: HCl buffer (pH 1.2), PBS (pH 7.4) pH 7.4: from 80%/2 h to 100%/15 min (different formulations) pH 1.2: 5–20% released in 2 h	Pharma	[93]
MCC + PVP	Curcumin	Supercritical Anti-solvent + Fluidized Bed	Microparticles	Size: ca. 140 µm Morphol.: spherical Excellent flow properties	Solvent: 0.25 % *w/v* SDS Without PVP: 50%/1 h With PVP 100%/5 min	Pharma	[94]

Legend: MCC—Microcrystalline Cellulose; SPC—Soya Phosphatidylcholine; PVP—Poly (vinyl pyrrolidone); Morphol.—Morphology; TG—Thermal Degradation temperature; EE—Encapsulation Efficiency; ZP—Zeta Potential; PBS—Phosphate-Buffered Saline; SDS—Sodium Dodecyl Sulphate.

**Table 3:** Studies on the use of cellulose nanocrystals for the encapsulation and controlled release of liposoluble compounds.

Encapsulating Material(s)	Active Ingredient(s)	Method	Final Structure	Encapsulation Results	Release Results	Application	Reference
CNC modified with CTBA	Docetaxel, Paclitaxel and Etoposide	Incubation	Nanocomplexes	EE (DTX, PTX) = 90% EE (ETOP) = 48%	Solvent: PBS (pH 7.4) Rapid release: 20%/1 h DTX: 59%/2 d PTX: 44%/2 d ETOP: 75%/2 d	Pharma	[18]
CNC + Chitosan	Curcumin	Layer-by-Layer assembly	Multilayer (*n* = 10) films Multilayer (*n* = 5) microcapsules	LC: 1.74 μg/cm Morphology: porous, nanofibrous	Solvent: PBS (pH 7.4) Rapid release: 35%/1 h 65% released/8 h Release kinetics: Korsmeyer model 0.22 release exponent	Pharma	[15]
CNC + Cationic cyclodextrins	Curcumin	Electrostatic coupling + Incubation	Nanocomplexes	LC = 8–10% ZP: −30 mM	Solvent: H_2_O/CHCl_3_ Rapid release: 15%/1 h 20–25%/8 h Enhanced antiproliferative effect on colorectal and prostatic cancer cell lines	Pharma	[95]
CNC + Chitosan	Curcumin	Swelling equilibrium	Hydrogel	EE: 41% Morphology: interconnected, porous Swelling ratio: 438%	Solvent: simulated gastric medium Prolonged release phase at 2.5 h (0.70 mg/L)	Pharma	[179]
CNC + PLGA	Curcumin	Electrospinning	Composite nanofibers	Size: 100–200 nm wide	Solvent: PBS (pH 7.4) 74%/1 d; 90%/6 d Bioactivity of Cur preserved Excellent biocompatibility	Pharma	[183]
CNC + Collagen as scaffold Gelatin as carrier	Curcumin	Emulsion solvent evaporation + Freeze-Drying	Scaffolds containing curcumin-loaded microspheres	Morphology: interconnected, porous Pore size: 80–110 μm Porosity: 90%	Solvent: DTM solution 35%/1 d 100%/10 d	Pharma	[184]
CNC modified with CTBA	Curcumin	Incubation	Nanocomplexes	EE (unmodified CNC) = 27% EE (CTBA-CNC) = 80–96%	-	Pharma	[39]
TEMPO-oxidized CNC (TOCNC) + HPβCD + PEG200	Curcumin Carvacrol	Casting + Impregnation	Films	Loading of carvacrol and curcumin increased compared with virgin TOCNC	Solvent: distilled water Curcumin: 95–100%/2 h Carvacrol: 90–100%/2 h TOCNC/HPβCD loading carvacrol exhibited excellent antibacterial activities	Food packaging	[178]
CNC modified with TA and DA	Curcumin	Incubation	Nanocomplexes	EE (unmodified CNC) = 8–54% EE (TA-DA-CNC) = 95–99%	-	Pharma	[5]
Aminated-CNC + Chitosan + Aminated-Graphene + synthetic dialdehyde	Curcumin	Schiff base reaction	Hydrogel	Morphology: cross-linked, porous Swelling ratio: 6985%	Solvent: PBS (pH 7.4 and 5.4) pH 7.4: 25%/12 h pH 5.4: 55%/12 h Fast gelation in rat’s skin by subcutaneous injections Antibacterial activity against gram-positive bacteria	Pharma	[180]
CNC modified with CTBA	Luteolin Luteoloside	Incubation	Nanocomplexes	LC (luteolin) = 12.9 mg/g LC (luteoloside) = 56.9 mg/g ZP: ca. −30 mV	Solvent: PBS (pH 7.4 and 6.4) Luteolin pH 7.4: 57%/24 h pH 6.4: 44%/24 h Luteoloside pH 7.4: 72%/24 h pH 6.4: 57%/24 h	-	[114]
Magnetic CNC + Alginate	Ibuprofen	Co-precipitation + Extrusion into a CaCl_2_ gelation bath	Hydrogel beads	EE = 38% LC = 3.2% Size: 2.3–2.4 nm (wet), 1.9–2.0 mm (freeze dried) Morphology: ellipsoidal, wrinkled Swelling ratio: 1878–2477%	Solvent: PBS (pH 7.4) Rapid release: 45–60%/30 min 100%/5–6 h	Pharma	[29]
CNC, TOCNC or ACNC + Chitosan + TPP	Ketoprofen	Ionic gelation	Nanoparticles	EE = 73–79% ZP: ca. 30 mV Size: 195–235 nm Morphology: spherical PDI: 0.1–0.2	Solvent: PBS (pH 7.4) Rapid release: 20–50%/2 h CNC: 41–46%/6 h TOCNC: 58–62%/6 h ACNC: 60–64%/6 h	Pharma	[28]

Legend: CNC—Cellulose nanocrystals; CTBA—Cetyltrimethylammonium bromide; PLGA—Poly(lactic-co-glycolic acid); TOCNC—TEMPO-oxidized CNC; HPβCD—Hydroxypropyl-beta-cyclodextrin; PEG200—Polyethylene glycol 200; TA—Tannic acid; DA—Decylamine; ACNC—Aminated CNC; TPP—Pentasodium tripolyphosphate; DTX—Docetaxel; PTX—Paclitaxel; ETOP—Etoposide; EE—Encapsulation efficiency, LC—Loading capacity, ZP—Zeta potential; PDI—Polydispersity Index; PBS—Phosphate buffered saline, DTM solution—PBS (0.01 M, pH 7.4) + 0.5% Tween (*v/v*) + 3% methanol.

**Table 4:** Studies on the use of cellulose nanofibers for the encapsulation and controlled release of liposoluble compounds.

Encapsulating material(s)	Active ingredient(s)	Encapsulation Method	Final Structure	Encapsulation results	Release results	Application	Reference
CNF/CNC as water phase Spin-probe, IPDI, and dibutyltin dilaurate as oil phase TOCNF as matrix	Hexadecane	Direct mini-emulsion polymerization + Filtration through a hydrophobic membrane	Microcapsules containing several primary capsules in a CNF matrix	Size: Primary capsule = 1–2 μm Aggregate capsule = 6–11 μm Oxygen uptake rate was reduced for both capsules	-	Food, Pharma	[186]
CNF + Gum Arabic	Sweet orange essential oil	Sonication + Spray drying	Microparticles	LC: 17.0% Morphology: spherical, wrinkled TG: 323 °C	-	Food	[115]
CNF	Ibuprofen	Sonication + Spray drying	Microparticles	LC: 1.7% Morphology: fibrous, spheroid Size: ca. 5 µm	Solvent: PBS (pH 7.4) Slow-release rate over 2 months	Pharma	[118]
CNF	Furosemide	Casting + Drying	Nanofoams	LC: 21%, 50% Size: 0.4–0.8 mm thick Density: ca. 0.035 g/cm^3^ Porosity: 98%	Solvent: simulated gastric fluid (pH 1.6) Rapid release of ca.25%/2 h 50% wt foam: 45%/24 h 21% wt foam: 65%/24 h	Pharma	[150]
HFBI as coating CNF + Trehalose as matrix	Itraconazole	Anti-solvent precipitation + Freeze-drying	Immobilized particles in CNF matrices	Particle size: ca.100 nm CNFs played a critical effect on the stabilization of the particles (storage for more than ten months)	Solvent: NaCl/HCl solution (pH 1.2) Rapid release of ca. 60%/10 min Before storage: 90%/90 min After 12 weeks storage: 75%/90 min	Pharma	[144]
CNF	Itraconazole	Sonication + Drying	Films	EE: >80% LC: 17–40%	Solvent: NaCl/HCl solution (pH 1.2) 55–90%/80 d Zero-order release kinetics	Pharma	[149]

Legend: CNF—Cellulose nanofibers; CNC—Cellulose nanocrystals; Spin-probe—Methyl 16-doxyl-stearate; IPDI—Isophorone diisocyante; TOCNF—TEMPO-oxidized CNF; HFBI—Hydrophobin; LC—Loading Capacity; TG—Thermal Degradation temperature; EE—Encapsulation Efficiency; PBS—Phosphate Buffered Saline.
